# An Investigation of the Electrical Performance of Polymer-Based Stretchable TFTs Under Mechanical Strain Using the Y-Function Method

**DOI:** 10.3390/polym18030419

**Published:** 2026-02-05

**Authors:** Hyunjong Lee, Hyunbum Kang, Chanho Jeong, Insung Choi, Sohee Kim, Eunki Baek, JongKwon Lee, Dongwook Kim, Jaehoon Park, Gae Hwang Lee, Youngjun Yun

**Affiliations:** 1 School of Semiconductor·Display Technology, Hallym University, Chuncheon 24252, Republic of Korea; hyunjong9951@gmail.com (H.L.); jbird97@naver.com (C.J.); gic0124@gmail.com (I.C.); sirasohee@gmail.com (S.K.); dmsrl426@gmail.com (E.B.); nujcog@naver.com (J.L.); d.kim@hallym.ac.kr (D.K.); jaehoonpark@hallym.ac.kr (J.P.); 2 Department of Chemistry, University of Ulsan, Ulsan 44776, Republic of Korea; hbkang@ulsan.ac.kr; 3 Samsung Advanced Institute of Technology (SAIT), Samsung Electronics, Suwon 16678, Republic of Korea

**Keywords:** organic thin-film transistors (OTFTs), stretchable transistors, Y-function method

## Abstract

Stretchable semiconductors capable of maintaining electrical performance under large mechanical deformation are essential for reliable wearable electronic devices. However, polymer semiconductors often suffer from electrical degradation when subjected to tensile strain. In this study, electrical stability under strain was achieved by using a rubber-blended poly(2,5-bis(2-octyldodecyl)-3,6-di(thiophen-2-yl)diketopyrrolo[3,4-c]pyrrole-1,4-dione-alt-thieno[3,2-b]thiophene) (DPPT-TT) polymer semiconductor based on a conjugated polymer/elastomer phase separation-induced elasticity (CONPHINE) structure. Unlike most previous studies on fully stretchable thin-film transistors (TFTs), which primarily report overall performance changes under mechanical strain, this work systematically identifies the dominant origin of electrical performance degradation through a stepwise electrical analysis encompassing the gate insulating layer, the semiconductor layer, and complete devices. Bottom-gate top-contact (BGTC) and bottom-gate bottom-contact (BGBC) devices were fabricated on rigid Si/SiO_2_ substrates to examine the intrinsic properties of the DPPT-TT/styrene-ethylene-butylene-styrene (SEBS) CONPHINE film. As a result, the device exhibits 90% mobility retention even at 100% tensile strain applied parallel to the charge transport direction. Quantitative resistance analysis using the Y-function method reveals that variations in channel resistance play a dominant role in strain-induced performance degradation, whereas changes in contact resistance contribute only marginally. These findings demonstrate that stabilizing channel resistance, rather than contact resistance, is important for achieving high mobility retention under large mechanical deformation, thereby providing concrete and quantitative design guidelines for reliable stretchable TFTs.

## 1. Introduction

Flexible and stretchable electronic devices have emerged as key technology enabling the development of new applications such as wearable healthcare systems, electronic skin, and biomedical sensors [1,2,3,4,5]. In these applications, electronic components are repeatedly subjected to mechanical deformation, which makes mechanical compliance and electrical stability equally critical. Stretchable semiconductors that can sustain charge transport under large strain are therefore indispensable for the realization of mechanically reliable wearable electronics [6,7,8,9]. Polymer semiconductors are particularly attractive for stretchable electronics owing to their intrinsic mechanical flexibility and solution processability [4,10,11]. Various strategies have been proposed to impart stretchability to polymer semiconductors, including rubber blending, dynamic bonding, and mechanically guided structural designs [12,13,14,15]. Among these approaches, blending conjugated polymers with elastomers has proven effective in achieving intrinsic stretchability while preserving electrical performance. In particular, the CONPHINE concept enables the formation of nanoconfined semiconductor domains that maintain interconnected charge transport pathways even under large deformation [4,8,9,15,16,17,18,19,20]. Although numerous studies have reported TFTs with high electrical stability under mechanical strain, most prior work has primarily focused on overall device performance or material-level stretchability. In contrast, quantitative analysis that clearly distinguishes the respective contributions of the gate insulator, semiconductor channel, and contact resistance under identical deformation conditions remains limited. As a result, the dominant physical origin of strain-induced electrical performance degradation in fully stretchable TFTs is still not well understood. In this study, we address this gap by systematically analyzing the electrical behavior of polymer-based stretchable TFTs using a stepwise device architecture approach combined with Y-function analysis. By independently evaluating the gate insulating layer, the semiconductor layer, and the fully stretchable TFT devices, we quantitatively separate the contributions of channel resistance and contact resistance under mechanical strain. This approach enables the identification of the dominant degradation mechanism governing electrical performance in fully stretchable polymer TFTs.

## 2. Materials and Methods

### 2.1. Materials

**Figure 1:**
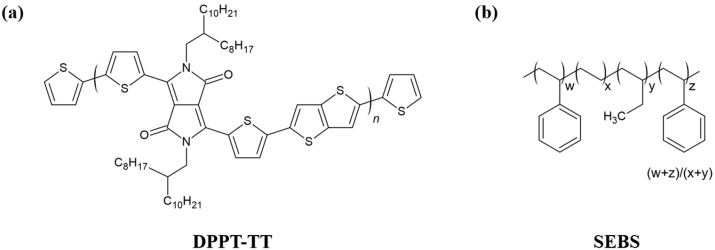
Chemical structures of (**a**) DPPT-TT and (**b**) SEBS.

DPPT-TT was employed as a semiconducting material, and its chemical structure is shown in Figure 1a [4,8,17]. The elastomeric component SEBS, shown in Figure 1b, was used for both semiconductor rubber blending and as a stretchable substrate material. SEBS H1221 (Asahi Kasei, Tokyo, Japan) was utilized for the semiconductor layer and substrate, whereas SEBS H1052 (Asahi Kasei) was used to fabricate the dielectric layer.

SEBS H1221 has a styrene/ethylene-butylene (S/EB) weight ratio of 12/88 (*w*/*w*) and a Young’s modulus of approximately 1.5 MPa, whereas SEBS H1052 has an S/EB weight ratio of 20/80 (*w*/*w*) and a higher Young’s modulus of approximately 5.0 MPa [6,7,9,21,22]. Chlorobenzene (anhydrous, Sigma-Aldrich, Saint Louis, MO, USA) was used as the solvent to prepare the DPPT-TT/SEBS solutions. Octadecyltrichlorosilane (OTS; Acros Organics, Geel, Belgium) was employed to modify the SiO_2_ surface and render it hydrophobic prior to spin coating for CONPHINE film formation [23]. Poly(dimethylsiloxane) (PDMS) (Sylgard 184, Dow Corning, Midland, MI, USA) was used in the stamp-and-transfer process [21,22].

### 2.2. Method

DPPT-TT and SEBS H1221 were dissolved in chlorobenzene at a weight ratio of 3:7 (*w*/*w*) to prepare the semiconductor solution. In previous studies using the same polymer semiconductor DPPT-TT, the DPPT-TT:SEBS mixing ratio of 3:7 was reported to provide an optimal balance between electrical performance and stretchability [16]. Therefore, the same ratio was adopted in this study. The solution was stirred overnight at 120 °C using a magnetic stirrer to ensure complete dissolution. The resulting solution was spin-coated onto an OTS-treated Si/SiO_2_ substrate to form a CONPHINE semiconductor film, which was subsequently used for the transfer process.

To fabricate BGTC devices, a DPPT-TT/SEBS (3:7) solution was spin-coated onto an OTS-treated Si/SiO_2_ substrate to form a CONPHINE semiconductor layer. The coated semiconductor film was annealed at 180 °C for 1 h under a nitrogen atmosphere to promote film formation and phase separation. The Au source and drain electrodes were thermally evaporated onto the semiconductor layer to a thickness of 50 nm, at a deposition rate of 1.0 Å/s.

To fabricate a device with a BGBC device, Au electrodes were first thermally evaporated onto an OTS-treated Si/SiO_2_ substrate to a thickness of 50 nm at a deposition rate of 1.0 Å/s. Subsequently, the DPPT-TT/SEBS semiconductor solution was spin-coated onto the patterned substrates to form the CONPHINE semiconductor layer. The coated films were annealed at 180 °C for 1 h under a nitrogen atmosphere.

To fabricate the insulating film, a glass substrate was plasma-treated (50 W, 60 s) and then spin-coated with a dextran solution (10 wt% in deionized water) at 1500 rpm for 20 s to form a water-soluble sacrificial layer. An SEBS H1052 dielectric layer (60 mg/mL in toluene) was subsequently spin-coated at 1000 rpm. The substrate was immersed in deionized water to dissolve the dextran layer, after which the free-standing insulating films were mechanically stretched and transferred onto glass substrates with pre-deposited Al bottom electrodes. The Al top electrodes were then deposited onto the transferred insulating films to enable electrical characterization. The capacitance per unit area and breakdown voltage were measured using an HP 4192A LF impedance analyzer (Hewlett-Packard, Santa Rosa, CA, USA) and a semiconductor parameter analyzer (ELECS-421C, ELECS, Seoul, Republic of Korea).

To evaluate the electrical performance of the semiconductor film after stretching, Si/SiO_2_ substrates with bottom source-drain electrodes were fabricated by thermally evaporating 50 nm of Au at a deposition rate of 1.0 Å/s. The CONPHINE semiconductor film was then transferred onto the Au-patterned Si/SiO_2_ substrates using a PDMS stamp, which resulted in BGBC devices in which only the semiconductor layer was subjected to mechanical stretching.

**Figure 2:**
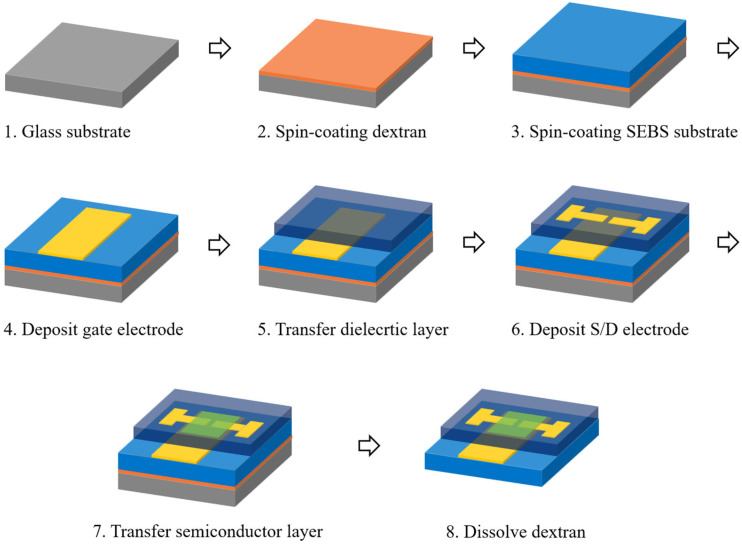
Fabrication process for fully stretchable TFTs.

**Figure 3:**
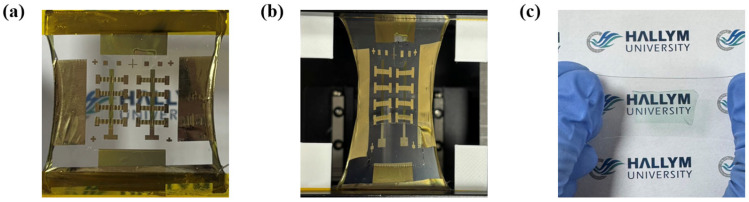
Images of fully stretchable TFTs (**a**) before strain and (**b**) after 100% strain. (**c**) Image of the semiconductor film on PDMS after 100% strain.

The process used to fabricate the fully stretchable TFTs is illustrated in Figure 2. A water-soluble sacrificial dextran layer was first formed on plasma-treated glass substrates (50 W, 60 s) by spin coating at 1500 rpm for 20 s, followed by annealing at 180 °C for 20 min. SEBS H1221 (150 mg/mL) was then spin-coated onto the dextran layer at 1000 rpm for 60 s and annealed at 150 °C for 60 min to form a stretchable substrate layer. A microcracked Au gate was subsequently deposited by thermal evaporation to a thickness of 75 nm (0.1 Å/s). The insulating layer, prepared by spin coating SEBS H1052 at 1000 rpm, was transferred onto the gate electrode using a PDMS stamp (10:1 mixing ratio). Au source-drain electrodes were then deposited to a thickness of 75 nm (0.1 Å/s). The CONPHINE semiconductor film was transferred onto the source/drain electrodes using a PDMS stamp to complete the fully stretchable TFT structure. The PDMS thin film with the delaminated semiconductor layer was laminated onto the target substrate, followed by vacuum degassing for 5 min to remove trapped air at the interface. The PDMS layer was then peeled off, leaving only the transferred semiconductor thin film on the substrate. After the transfer process, the sample was annealed at 90 °C for 60 min in a vacuum oven. The dextran sacrificial layer was dissolved in deionized water, and the devices were dried in a vacuum oven at room temperature to remove residual moisture prior to the electrical measurements. Representative images of the fully stretchable TFTs before and after mechanical deformation are shown in Figure 3 along with the stretched semiconductor film on PDMS. The surface morphology of the semiconductor film was characterized using atomic force microscopy (AFM) operated in tapping mode (Jupiter XR, Oxford Instruments Asylum Research, Santa Barbara, CA, USA) with a scan setpoint of 50% of the free oscillation amplitude and a scan rate of 0.75 Hz and electrical measurements were performed using a semiconductor parameter analyzer (ELECS-421C, ELECS, Seoul, Republic of Korea). The channel and contact resistance values were extracted using the Y-function method as described in Equations (1) and (2) [24,25,26,27].

(1) $$g_{m}=\frac{\partial I_{\;d}}{\partial V_{g}},\;Y=\frac{I_{\;d}}{\sqrt{g_{m}}},\;S_{1}=\mathrm{slope}\left(Y,{\;V}_{g}\right),\;S_{2}=\mathrm{slope}\left(\frac{1}{\sqrt{g_{m}}},{\;V}_{g}\right),$$

(2) $$R_{c}=\frac{S_{2}}{S_{1}}V_{d},{\;R}_{\mathrm{ch}}=L/(\mu_{0}C_{i}W\left(V_{g}-V_{\mathrm{th}}\right)).$$

## 3. Results and Discussion

### 3.1. Y-Function Method

To quantitatively analyze strain-induced variations in electrical performance, the channel and contact resistances were extracted using the Y-function method (YFM). The Y-function method is based on the drift–diffusion transport model and assumes operation in the linear regime with gate-voltage-independent contact resistance. In TFTs, however, mechanical deformation can modify both carrier mobility and contact conditions, potentially affecting the validity of these assumptions. To address this limitation, a stepwise analytical approach was adopted in this study to decouple the electrical contributions of the gate insulator, semiconductor layer, and complete devices, thereby minimizing ambiguity in the interpretation of YFM-derived parameters.

Alternative extraction techniques such as the transmission line method (TLM) require multiple devices with different channel lengths. In stretchable TFT systems, where geometric deformation occurs simultaneously with mechanical strain, maintaining identical strain conditions across devices is challenging. In contrast, the Y-function method enables the extraction of channel and contact resistances from the transfer characteristics of a single device, providing a consistent and practical basis for comparative analysis under mechanical strain.

### 3.2. Untransferred Device

**Figure 4:**
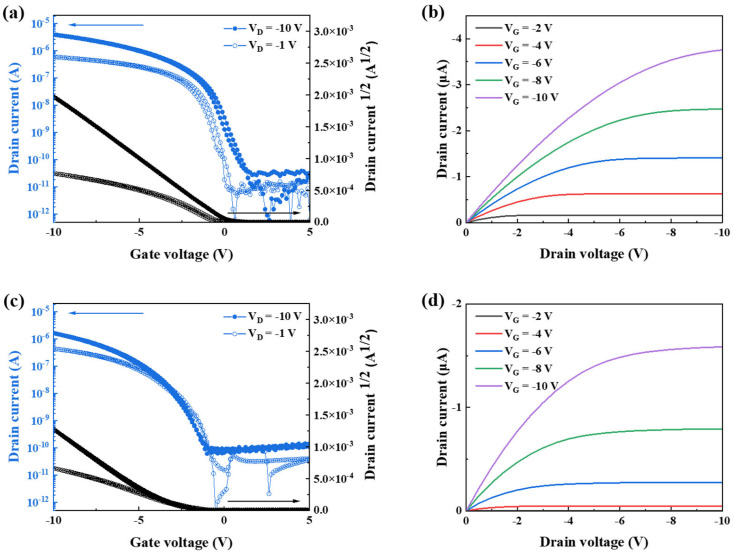
(**a**) Transfer curves (V_D_ = −1, 10 V) of the BGTC structure. (**b**) Output curves of the BGTC structure. (**c**) Transfer curves (V_D_ = −1, 10 V) of the BGBC structure. (**d**) Output curves of the BGBC structure.

Figure 4a–d show the electrical characteristics of the spin-coated devices fabricated to evaluate their intrinsic transistor performance. Both BGTC and BGBC structures were employed, and their charge transport properties were systematically compared. For the BGTC devices, the extracted linear and saturation mobilities were 0.620 (±0.021) cm^2^ V^−1^ s^−1^ and 0.692 (±0.024) cm^2^ V^−1^ s^−1^, respectively. In comparison, the BGBC devices exhibited linear and saturation mobilities of 0.660 (±0.010) cm^2^ V^−1^ s^−1^ and 0.621 (±0.014) cm^2^ V^−1^ s^−1^, respectively. The comparable mobility values obtained from the two device architectures indicate that contact configuration exhibited a negligible influence on the intrinsic charge transport properties of the spin-coated DPPT-TT/SEBS CONPHINE films.

### 3.3. Gate Insulator

**Figure 5:**
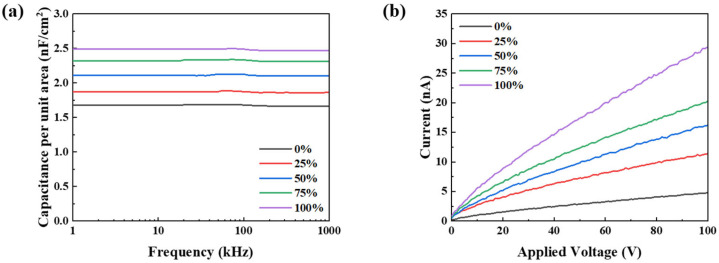
(**a**) Capacitance per unit area of the SEBS dielectric layer under strain. (**b**) Breakdown voltage of the SEBS dielectric layer under strain.

**Figure 6:**
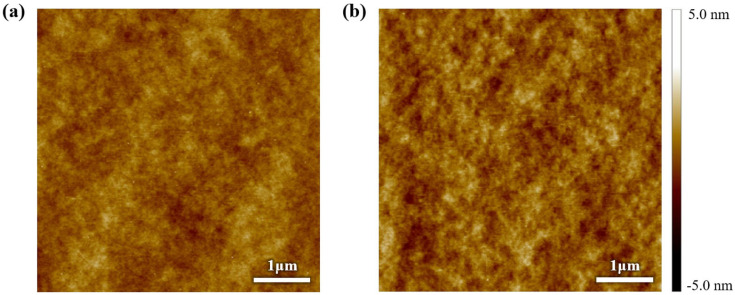
5 × 5 μm^2^ AFM height image of the SEBS dielectric layer (**a**) before strain and (**b**) after 100% strain. The RMS roughness increases from 0.397 nm to 0.524 nm.

When tensile strain was applied to the insulating film, the capacitance per unit area decreased linearly with increasing strain, as shown in Figure 5a. This behavior is attributed to the effective decrease in dielectric thickness induced by mechanical elongation. Notably, no dielectric breakdown was observed even at 100% tensile strain under an applied voltage of up to 100 V (Figure 5b), which indicates the excellent electrical robustness of the insulating layer. An analysis with AFM revealed only a slight increase in the roughness of the surface after stretching, with the root-mean-square (RMS) roughness increasing from 0.397 to 0.526 nm at 100% strain (Figure 6).

### 3.4. Semiconductor Film

**Figure 7:**
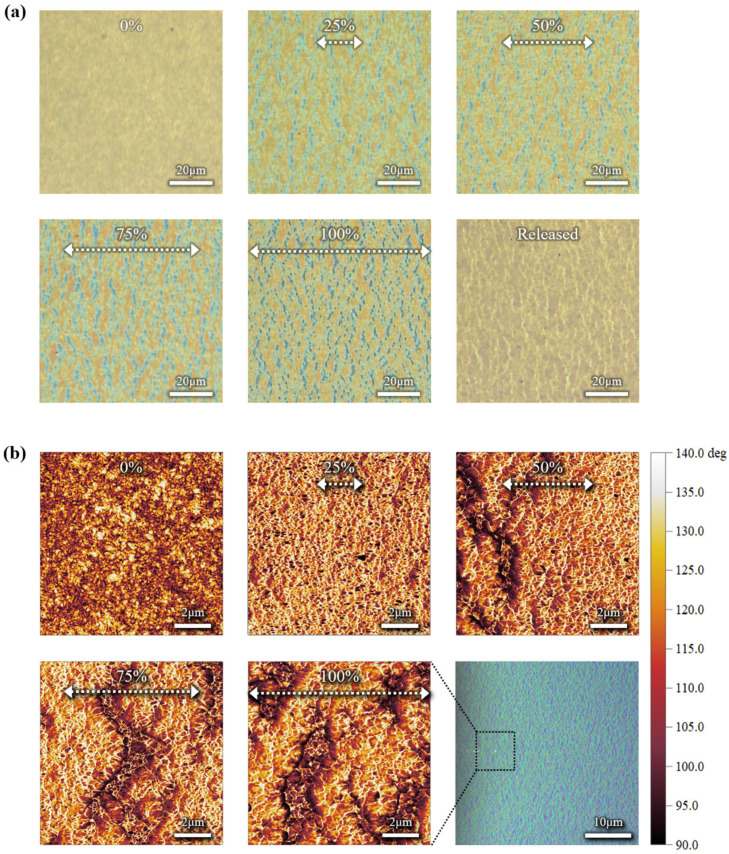
(**a**) Optical microscopy image and (**b**) 5 × 5 μm^2^ AFM phase image of the CONPHINE film under uniaxial tensile strain applied along the horizontal direction, as indicated by the arrows, with the applied strain values shown at the top of each image, where (**a**) shows that the spacing between microcracks increases with increasing strain, and (**b**) shows that the dark regions correspond to SEBS and the bright regions correspond to conjugated polymer chains, with increasing separation between polymer domains with increasing strain.

**Figure 8:**
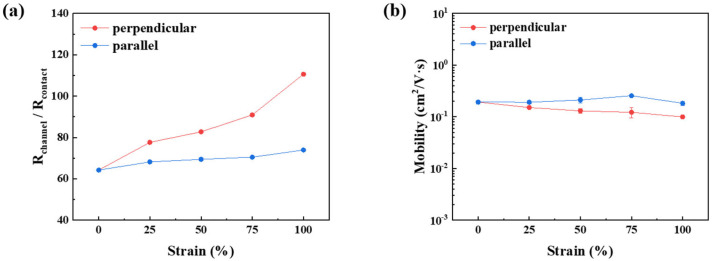
(**a**) Channel/contact resistance ratio and (**b**) mobility of the semiconductor film under tensile strain applied only to the semiconductor layer. Error bars represent the standard deviation obtained from measurements of more than five independent devices.

The CONPHINE semiconductor film maintained interconnected charge transport pathways through the nanoconfined polymer chains embedded within the elastomer matrix, as shown in Figure 7a,b [4,8,16]. While alternative strategies such as mechanically guided structural designs, dynamic coupling, or engineered electrode architectures can effectively accommodate large mechanical deformation, these approaches often introduce additional structural variables that complicate quantitative electrical analysis. In contrast, the CONPHINE structure provides intrinsic stretchability at the material level through nanoconfined semiconductor domains embedded within an elastomer matrix. In the CONPHINE structure, mechanical deformation is preferentially accommodated by the elastomer-rich phase, while the phase-separated conjugated polymer domains remain mechanically constrained yet electrically connected. This structural decoupling suppresses stress concentration within the semiconducting domains and prevents the formation of electrically inactive gaps during stretching, thereby preserving continuous charge percolation pathways under large tensile strain. Accordingly, the CONPHINE structure is particularly well suited for the primary objective of this study, namely, the quantitative identification of strain-induced changes in channel and contact resistance under identical deformation conditions. Degradation in mobility under mechanical stretching remained relatively limited owing to this structural feature. AFM analysis confirmed a gradual widening of the spacing between semiconductor domains upon stretching, which is consistent with the elastic deformation of the surrounding elastomer. The AFM phase images clearly indicate a continuous and well-connected semiconducting network embedded within the elastomeric matrix. This morphology-based interpretation is consistent with the high mobility retention observed under mechanical strain. When tensile strain was applied perpendicular to the charge transport direction, mobility decreased from 0.193 (±0.015) cm^2^ V^−1^ s^−1^ in the unstretched state to 0.100 (±0.003) cm^2^ V^−1^ s^−1^, primarily due to an increase in channel resistance. In contrast, when strain was applied parallel to the charge transport direction, the mobility remained relatively stable at 0.183 (±0.016) cm^2^ V^−1^ s^−1^. This pronounced anisotropic strain response originates from differences in how mechanical deformation disrupts charge transport pathways relative to the transport direction. Tensile strain applied perpendicular to the charge transport direction more severely perturbs π–π stacking and increases the separation between conjugated polymer domains, leading to enhanced energetic disorder and reduced carrier mobility. In contrast, strain applied parallel to the transport direction largely preserves effective percolation pathways, as polymer chains and interconnected domains remain preferentially aligned along the current flow direction, resulting in higher mobility retention. This behavior indicates that partial alignment of polymer chains along the charge transport direction compensated for the strain-induced increase in channel resistance, as shown in Figure 8a,b. The overall mobility of the transferred semiconductor devices was lower than that of the spin-coated devices. This reduction is mainly attributed to increased contact resistance, which is likely due to partial damage to the semiconductor film during the transfer process and incomplete electrical contact at the electrode-semiconductor interface.

### 3.5. Fully Stretchable TFTs

**Figure 9:**
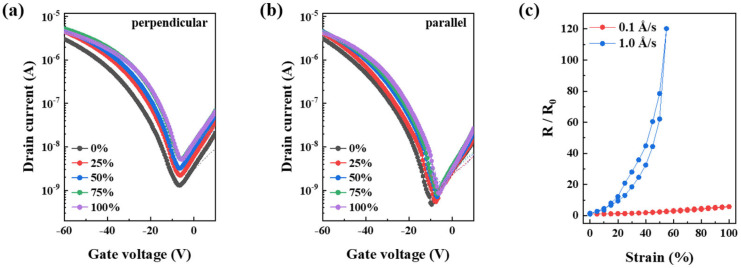
Transfer curves (V_D_ = −60 V) under (**a**) perpendicular and (**b**) parallel strain with respect to the charge transport direction. (**c**) Stretch stability of microcracked Au electrodes at different deposition rates (0.1 Å/s, 1.0 Å/s). The fully stretchable TFTs operate stably under tensile strain, and the normalized resistance (R/R_0_) represents the resistance change relative to the initial unstretched state.

**Figure 10:**
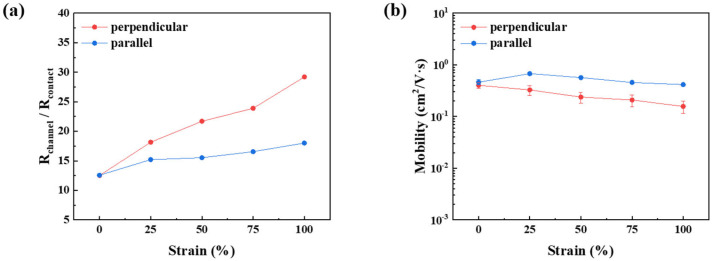
(**a**) Channel/contact resistance ratio and (**b**) mobility of the fully stretchable TFT under tensile strain. Error bars represent the standard deviation obtained from measurements of more than five independent devices.

Figure 9a,b show the transfer characteristics of fully stretchable TFTs measured under different tensile strain conditions. To ensure the mechanical and electrical stability of the electrodes during large deformation, microcracked Au electrodes deposited at different rates were evaluated as summarized by the normalized resistance (R/R_0_) in Figure 9c. Electrodes deposited at a higher rate of 1.0 Å/s exhibited a rapid increase in resistance and electrical failure at strains exceeding approximately 55%, whereas those deposited at 0.1 Å/s maintained continuous conductive pathways up to 100% strain. When tensile strain was applied perpendicular to the charge transport direction, the mobility of fully stretchable TFTs decreased from 0.461 (±0.052) cm^2^ V^−1^ s^−1^ in the unstretched state to 0.157 (±0.043) cm^2^ V^−1^ s^−1^ at 100% strain, primarily due to a pronounced increase in channel resistance. In contrast, when strain was applied parallel to the charge transport direction, channel resistance variation was limited, and the mobility remained relatively stable at 0.415 (±0.012) cm^2^ V^−1^ s^−1^. This directional dependence indicates that strain-induced alignment of the semiconductor pathways effectively mitigates the degradation of channel resistance, which is consistent with the trends shown in Figure 10a,b.

The combined effects of a strain-induced increase in channel resistance and a reduction in contact resistance due to an improved semiconductor-insulator interface resulted in a significantly lower channel/contact resistance ratio in fully stretchable TFTs compared to devices in which only the semiconductor layer was stretched, as shown in Figure 8a and Figure 10a. Despite differences in device architecture, both configurations exhibited a similar trend in the channel/contact resistance ratio, indicating that variations in channel resistance play a dominant role in determining the electrical characteristics of stretched TFTs, while changes in contact resistance have a comparatively minor impact. The improvement in the semiconductor-insulator interface was further confirmed by a substantial reduction in interface trap density when SEBS was employed as a dielectric material. Devices transferred onto conventional SiO_2_ gate dielectrics exhibited an interface trap density of 2.26 × 10^12^ cm^−2^ eV^−1^, whereas devices transferred onto SEBS dielectric layers showed a reduced trap density of 9.77 × 10^11^ cm^−2^ eV^−1^. As a consequence of the improved interfacial quality, the mobility in the unstretched state increased from 0.193 (±0.015) to 0.461 (±0.052) cm^2^ V^−1^ s^−1^. The error bars shown in Figure 8 and Figure 10 represent the standard deviation obtained from measurements of more than five independent devices. Although formal statistical hypothesis testing was not applied due to the limited sample size and inherent variability of stretchable device fabrication, the observed trends were highly consistent across devices and strain conditions. Therefore, the terms “dominant” and “minor” effects are used to describe relative contributions based on comparative magnitude and reproducibility rather than strict statistical significance.

To place the present results in the context of prior studies, Table 1 summarizes representative reports on stretchable polymer TFTs, focusing on key performance metrics including charge carrier mobility, mobility retention under tensile strain, maximum applied strain, device architecture, and resistance extraction methodology. Compared to previously reported CONPHINE-based and other intrinsically stretchable TFTs, the mobility retention of approximately 90% at 100% tensile strain achieved in this work is comparable to, or higher than, values reported for fully stretchable polymer TFT systems operating under similar strain levels. It should be noted, however, that direct quantitative comparison across studies is not always straightforward due to differences in experimental conditions. For example, several prior reports employ partially stretchable device architectures or mechanically guided electrode designs, whereas the present study focuses on fully stretchable BGBC architectures. In addition, deformation and testing modes vary significantly among studies, including differences in strain direction, strain application sequence, and measurement under static or cyclic loading. Another important distinction lies in the electrical analysis methodology. While many previous studies report overall device performance under strain, the present work employs a stepwise device architecture combined with the Y-function method to quantitatively separate the contributions of channel resistance and contact resistance under identical deformation conditions. This analytical approach enables direct identification of the dominant origin of strain-induced electrical performance degradation, which is not accessible through conventional device-level analysis alone. Taken together, these comparisons indicate that the electrical stability achieved in this study is competitive with representative literature values, while the primary contribution of this work lies in providing a consistent experimental framework for quantitatively isolating strain-induced degradation mechanisms in fully stretchable polymer TFTs.

## 4. Conclusions

In this study, we systematically investigated the degradation in the electrical performance of polymer-based fully stretchable TFTs under mechanical strain using a stepwise device analysis approach. The gate-insulating film exhibited stable electrical operation under tensile deformation, which indicates that tensile deformation was not a limiting factor in terms of reliability. For the CONPHINE semiconductor film, mobility strongly depended on the direction of the strain: when the film was stretched in a direction perpendicular to that of the charge transport, the mobility decreased from 0.193 to 0.100 cm^2^ V^−1^ s^−1^, whereas it remained relatively stable at 0.183 cm^2^ V^−1^ s^−1^ under parallel stretching. This behavior suggests that polymer chain alignment may stabilize charge transport pathways during mechanical deformation.

In fully stretchable TFTs, using SEBS as both the semiconductor elastomer matrix and the gate dielectric significantly improved the semiconductor–insulator interface and reduced the interface trap density from 2.26 × 10^12^ to 9.77 × 10^11^ cm^−2^ eV^−1^. As a result, the mobility in the unstretched state increased from 0.193 to 0.461 cm^2^ V^−1^ s^−1^. Under mechanical strain, mobility decreased to 0.157 cm^2^ V^−1^ s^−1^ when stretched perpendicular to the charge transport direction but remained stable at 0.415 cm^2^ V^−1^ s^−1^ under parallel stretching. The results of Y-function analysis showed that channel resistance variation was the dominant factor governing performance degradation in the fully stretchable TFTs studied, whereas the contribution of contact resistance was comparatively minor. The electrical stability achieved in this work is comparable to representative values reported in recent studies in stretchable polymer TFTs. Specifically, the fully stretchable TFTs exhibited a mobility retention of approximately 90% at 100% tensile strain, which is comparable to previously reported values for CONPHINE-based and other intrinsically stretchable polymer semiconductor systems. Notably, this level of strain tolerance was achieved without employing complex mechanically guided architectures, highlighting the effectiveness of material-level stretchability. These findings highlight the critical importance of controlling channel resistance through polymer chain alignment and interface engineering to develop mechanically robust stretchable TFTs. Thus, this work provides practical design guidelines to improve the mechanical reliability of stretchable electronic devices based on the studied system. Beyond summarizing the experimental findings, this study provides practical design insights for mechanically robust stretchable TFTs. The results indicate that stabilizing channel resistance is more critical than minimizing contact resistance for maintaining mobility under large tensile strain in the studied system. In CONPHINE-based systems, this can be achieved by preserving continuous charge percolation pathways through polymer chain alignment along the transport direction and stress accommodation by the elastomer-rich phase.

While the identified dominance of channel resistance stabilization provides a significant design principle for intrinsically stretchable polymer TFTs, it is important to define the boundary conditions of these findings. Our analysis was conducted using a specific material system (DPPT-TT/SEBS) and a BGBC architecture under single-cycle uniaxial strain. Therefore, the applicability of these conclusions to alternative geometries, different polymer–elastomer blends, or complex deformation modes—such as cyclic loading and biaxial strain—remains to be further explored. Future research will focus on validating this analytical framework across a broader range of materials and operational environments to ensure its general applicability.

## Figures and Tables

**Table 1:** Comparison with the reported intrinsically stretchable TFTs.

Semiconductor	Electrode	Dielectric	Device Structure	μ_0_ (0%) (cm^2^ V^−1^ s^−1^)	μ_100_ (100%) (cm^2^ V^−1^ s^−1^)	Maximum Strain (%)	Mobility Retention	Resistance Extraction Methodology	Refs.
DPPT-TT/SEBS	Au	SEBS	BGBC	0.461	0.415	100%	90%	YFM	This work
DPP-Polymer	CNT	PDMS	BGTC	0.6	-	100%	-	-	[28]
DPPT-TT/SEBS	CNT	SEBS	BGBC	0.59	0.55	100%	93%	TLM	[16]
P3HT/PDMS	PEDOT:PSS -LiTFSI	PDMS	BGTC	0.17	0.15	100%	88%	-	[29]
FT4-DPP/PEO	CNT/EGain	SEBS	TGBC	0.82	0.3	100%	37%	-	[30]
C12-DPP	CNT	SEBS	BGTC	0.463	0.1	100%	22%	-	[31]
CPP/DPP-TT	CNT	PDMS	BGTC	-	0.53	25%	-	TLM	[32]
DPPDTSE/SEBS	CNT	SEBS	BGBC	-	1.5	100%	-	TLM	[33]
DPP-TT/F4-TCNQ	CNT	PDMS	BGTC	1.03	-	100%	-	TLM	[34]
PII2TF/SEBS	PEDOT:PSS /PVA	PVA /PMAA	BGTC	0.068	0.053	100%	78%	-	[35]
DPPT-TT/iRUM	CNT	PDMS	BGTC	0.5	0.3	50%	60%	-	[36]
DPPT-TT/SEBS	Ag	SEBS	BGTC	0.288	0.183	100%	64%	TLM	[20]
PDVT-10/SEBS	CNT	SEBS	BGTC	2.74	2.53	100%	92%	-	[37]
PDPP-C4Ph	CNT	PDMS	BGTC	0.5	0.15	50%	30%	-	[38]

## Data Availability

The original contributions presented in this study are included in the article. Further inquiries can be directed to the corresponding authors.
